# A Ultrasensitive Near‐Infrared Fluorescent Probe Reveals Pyroglutamate Aminopeptidase 1 Can Be a New Inflammatory Cytokine

**DOI:** 10.1002/advs.201700664

**Published:** 2018-01-22

**Authors:** Qiuyu Gong, Ruifen Zou, Jie Xing, Lingchao Xiang, Renshuai Zhang, Aiguo Wu

**Affiliations:** ^1^ Key Laboratory of Magnetic Materials and Devices CAS & Key Laboratory of Additive Manufacturing Materials of Zhejiang Province & Division of Functional, Materials and Nanodevices Ningbo Institute of Materials Technology and Engineering Chinese Academy of Sciences No. 1219 ZhongGuan West Road Ningbo 315201 China; ^2^ University of Chinese Academy of Sciences Beijing 100049 China; ^3^ Key Laboratory of Experimental Marine Biology Institute of Oceanology Chinese Academy of Sciences Qingdao 266071 China

**Keywords:** inflammatory cytokine, near‐infrared fluorescent probes, pyroglutamate aminopeptidase 1

## Abstract

Previous study showed that pyroglutamate aminopeptidase 1 (PGP‐1) has a relationship with the immune response in cells. However, whether PGP‐1 is involved in inflammatory response in vivo and can serve as a new inflammatory cytokine are still unclear. To address these issues, a new near‐infrared fluorescent probe, which exhibits high selectivity and super sensitivity, is developed. With this probe, the up‐regulation of PGP‐1 (evidenced by western blot) in BALB/c mice legs and livers under the stimulation of two main immunopotentiators is revealed for the first time. The occurrence of inflammatory process (including tissue necrosis) in mice is determined by up‐regulation of tumor necrosis factor‐α and hematoxylin‐eosin staining. Interestingly, it is revealed for the first time that knocking down PGP‐1 leads to the weakness of inflammatory process in RAW264.7 cells. These new findings suggest that PGP‐1 is indeed involved in inflammatory response in vivo and can be a new inflammatory cytokine.

Inflammation, a protective response to damage factor that involves immune cells, blood vessels, and molecular mediators, plays a significant role in many diseases,^1^ on which considerable effort has already been expended.^2^ Pyroglutamate aminopeptidase 1 (PGP‐1, EC 3.4.19.3) is a type of enzyme that cleaves the peptide bond of pyroglutamic acid linked to the N‐terminal end of a protein, including some important antiinflammatory proteins like immunoglobulin,^3^ due to the fact that pyroglutamic acid is usually formed in N‐terminal end of immunoglobulin.[[qv: 3b]] This phenomenon reminds us of the relationship between PGP‐1 and inflammatory process. In a pioneering study by Ma and co‐workers,^4^ it has been showed that PGP‐1 has a relationship with the body's immune response in cell level and may serve as an indicator of cellular inflammatory response. However, whether PGP‐1 is involved in body's immune response in vivo and can serve as a new inflammatory cytokine are still unclear. To address these issues, herein, we develop a new near‐infrared (NIR) fluorescent probe by incorporating L‐pyroglutamic acid into the skeleton of hemicyanine fluorophore. Although the existing PGP‐1 probe has the advantage of high sensitivity,^4^ the relative short analytical wavelength (λ_ex/em_ = 585/625) makes it unsuitable to detect PGP‐1 in vivo. The new probe (**Figure** **1**a) exhibits not only long analytical wavelengths (λ_ex/em_ = 670/700 nm) but also high selectivity and super sensitivity to PGP‐1 under the physiological conditions. Using the probe as well as animal imaging device, the up‐regulation of PGP‐1 in BALB/c mice legs and livers under the stimulation of immunopotentiators is revealed for the first time, which is evidenced by Western blot (WB). And the occurrence of inflammatory process (including tissue necrosis) in mice is determined by up‐regulation of tumor necrosis factor‐α (TNF‐α: a common inflammatory factor) and hematoxylin‐eosin staining (H&E staining). Very interestingly, it is revealed for the first time that inhibiting PGP‐1 with small interfering RNA (siRNA) leads to the weakness of inflammatory process in RAW264.7 cells (weak up‐regulation of TNF‐α). These important findings as well as the previous results^4^ suggest that PGP‐1 is indeed involved in body's immune response and can be a new inflammatory cytokine.

**Figure 1:**
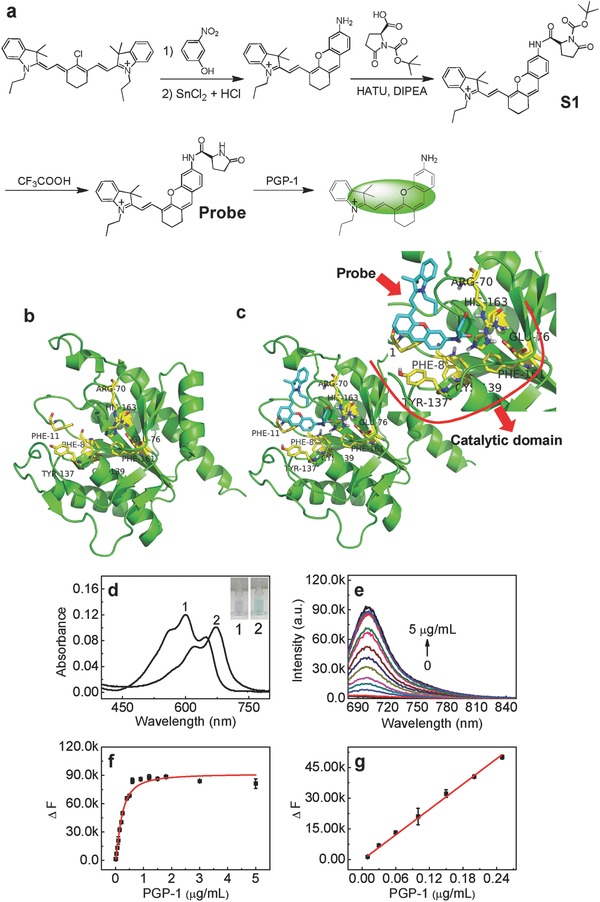
a) Synthesis of probe and its reaction with PGP‐1. b) Schematic drawing around the active site of PGP‐1 (PDB: 1iu8). The figure was produced with PyMOL. The related protein residues are shown in yellow. Glu‐76, Cys‐139, and His‐163 are three main catalytic sites; all the eight protein residues construct the catalytic domain. c) The binding mode of the probe with PGP‐1 (the inset is the magnified picture). d) Absorption spectra of probe (5 × 10^−6^ m) before (curve 1) and after (curve 2) reaction with PGP‐1 (5 µg mL^−1^) at 37 °C in 10 × 10^−3^ m phosphate buffer saline (PBS; pH 7.4). e) Fluorescence response of probe (5 × 10^−6^ m) to PGP‐1 at different concentrations (from bottom to top: 0–5 µg mL^−1^). f) The changes of fluorescence enhancement (∆*F*) of reaction systems with increase of PGP‐1 concentration. g) The plot of ∆*F* versus the PGP‐1 concentration. λ_ex/em_ = 670/700 nm.

We first prepared probe (Figure 1a) as an NIR fluorescent probe for PGP‐1. Hemicyanine fluorophore was chosen as a fluorescent skeleton due to its long analytical wavelength (λ_ex/em_ = 670/700 nm) and good biocompatibility,^5^ which would make it possible to detect the PGP‐1 in vivo. As shown in Figure 1a, the probe was synthesized as follows: hemicyanine fluorophore probe was prepared according to the previous method[[qv: 5b]] and then treating it with the protected L‐pyroglutamic acid, followed by removing the protecting group in the presence of trifluoroacetic acid (Figures S1–S4, Supporting Information). Moreover, the probe could be hydrolyzed by PGP‐1, which led to the recovery of fluorescence (Figure 1a). The molecule docking experiments show that there exist three main catalytic sites: His‐163, Glu‐76, and Cys‐139 in PGP‐1 protein (Figure 1b), which is consistent with previous result.^6^ The catalytic domain consists of the main protein residues: Arg‐70, His‐163, Glu‐76, Phe‐161, Cys‐139, Tyr‐137, Phe‐8, and Phe‐11.^6^ As shown in Figure 1c, the introduction of hemicyanine fluorophore has almost no effect on residence area of the L‐pyroglutamic acid in catalytic domain, which means the probe indeed can be hydrolyzed by PGP‐1.

Then, spectroscopic properties and analytical performance of probe were studied in detail. As shown in Figure 1d, probe shows a maximum absorption peak at about 600 nm; but after reaction with PGP‐1, the maximum absorption peak moves to about 670 nm, which makes it suitable to monitor PGP‐1 in vivo. Moreover, the probe exhibits extremely low background fluorescence; addition of PGP‐1, however, induces a dramatic change in the fluorescence spectra (Figure 1e,f). Under the optimized conditions (reaction for 25 min at 37 °C and pH 7.4; Figures S5 and S6, Supporting Information), the probe produces a good linear fluorescence off–on response to PGP‐1 in the concentration range of 0.01–0.25 µg mL^−1^ (Figure 1g), with an equation of ∆*F* = 206657 *C* (µg mL^−1^) – 338.37 (*R* = 0.998), where ∆*F* is the difference of fluorescence intensity of probe after and before reaction with PGP‐1. The detection limit^7^ is determined to be 0.18 ng mL^−1^ of PGP‐1, which is the lowerst as far as we know. Notably, the probe also exhibits rather high selectivity to PGP‐1 (Figures S7, Supporting Information). According to our hypothesis, the enzymatic cleavage reaction of the probe by PGP‐1 will cause the release of hemicyanine fluorophore (Figure 1a), and this hypothesis was verified by mass spectral analysis (*m*/*z* = 411.40 [M]^+^; Figure S8, Supporting Information). In addition, inhibitor experiments with iodoacetamide (Figure S9, Supporting Information) also supported that the fluorescence off–on response of the probe arises from the enzymatic action of PGP‐1. On the basis of Michaelis–Menten equation,^8^ the Michaelis constant (*K*_m_) of probe for PGP‐1 were determined to be 7 × 10^−6^ m (Figure S10a, Supporting Information), which is much lower than that of commercial probe (Figure S10b, Supporting Information), indicating probe has stronger affinity for PGP‐1. Moreover, the probe shows good biocompatibility (Figure S11, Supporting Information).

Next, we explore whether PGP‐1 has a relationship with inflammatory processes in living bodies by detecting the variation of PGP‐1 during the processes with our probe and WB analysis. As shown in **Figure** **2**a–d (after 20 min hypodermic injection of probe, the mice were placed to image; Figure S12, Supporting Information), the fluorescence intensities in nude BALB/c mice legs gradually increase with increasing the Freund's complete adjuvant (FCA: a common http://immunopotentiator 9) contents and treated time. This fluorescence increase reflects the up‐regulation of PGP‐1 in mice legs. Interestingly, we also observe that with the increase of FCA treatment time, the mice legs become more oedematous, which indicate the inflammatory processes in mice legs enhance (Figure 2e). Importantly, the up‐regulation of PGP‐1 and TNF‐α (its up‐regulation is related to the enhancement of inflammatory process^10^) are clearly confirmed by WB analysis (Figure 2f), which suggest the reliability of the new probe and the relativity between up‐regulation of PGP‐1 and inflammatory processes in living bodies.

**Figure 2:**
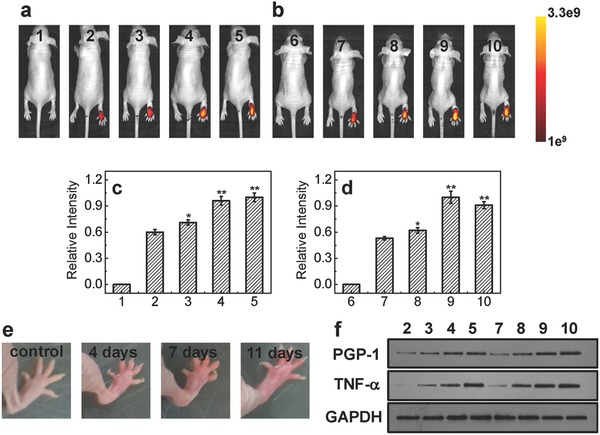
a,b) The fluorescence images in mice legs (1, 6: mice only; 2–5: mice were first hypodermic injected with different contents of FCA in legs, 0 (control), 25, 50, 100 µL, respectively, and then fed with 7 d; 7–10: mice were first hypodermic injected 100 µL of FCA in legs, and then fed with 0 (control), 4, 7, 11 d, respectively. After these, mice were hypodermic injected with 50 µL of probe (50 × 10^−6^ m) to image). λ_ex/em_ = 670/710 nm. c,d) The relative fluorescent intensities of above corresponding mice legs (the maximum fluorescent intensity was determined to be 1, *p < 0.01, **p < 0.001, as compared with the control, two‐sided Student's *t*‐test). e). The digital photographs of mice legs injected with 100 µL of FCA, and then fed with 0, 4, 7, and 11 d, respectively. f) The western blot analysis of PGP‐1 and TNF‐α in the above corresponding mice legs (the molecular weights of PGP‐1 and TNF‐α are determined to be 23 kDa and 26 kDa, respectively; glyceraldehyde‐3‐phosphate dehydrogenase, GAPDH, as a protein standard).

In order to further verify the relativity between up‐regulation of PGP‐1 and inflammatory processes in living bodies, the liver inflammation model (one common model) experiments were performed. Mice were first intraperitoneal injected with lipopolysaccharides/D‐galactosamine[[qv: 2a]] (LPS/D‐Gal) and then prepared to image. As shown in **Figure** **3**a (after 6 min intravenous injection of probe in the tail vain, the mice were placed to image; Figure S13, Supporting Information), the fluorescence in mice epigastrium is much stronger in LPS/D‐Gal treated group than that of control group (intraperitoneal injected with phosphate buffer saline (PBS)), which suggests the PGP‐1 in livers of LPS/D‐Gal treated group up‐regulates. It is clear that the fluorescent intensities in livers of treated mice with LPS/D‐Gal are higher than those of control group (Figure 3b,c), which means the LPS/D‐Gal indeed leads to the up‐regulation of PGP‐1 in livers. Importantly, the up‐regulation of PGP‐1 and TNF‐α of above samples are clearly confirmed by WB analysis (Figure 3d). Meanwhile, it can be seen from Figure 3e that the occurrence of livers inflammation (including tissue necrosis) in 16 h LPS/D‐Gal treated group (black arrow) and very slight inflammation (including tissue necrosis) in 2 h LPS/D‐Gal treated group are determined by H&E staining (As shown by the black arrow, there exist extensive necrosis of the hepatocytes around the central vein, deep cytoplasm of necrotic cells, and pyknosis or fragmentation of the nucleus.). At the same time, it should be noted that no inflammations or tissue necrosis in kidney areas are founded, so the fluorescences in kidney areas can be attributed to the endogenous expression of PGP‐1 or the metabolism of hemicyanine fluorophore (Figure 3b–d). These interesting findings further confirm that PGP‐1 has a strong relationship with inflammatory processes in living bodies.

**Figure 3:**
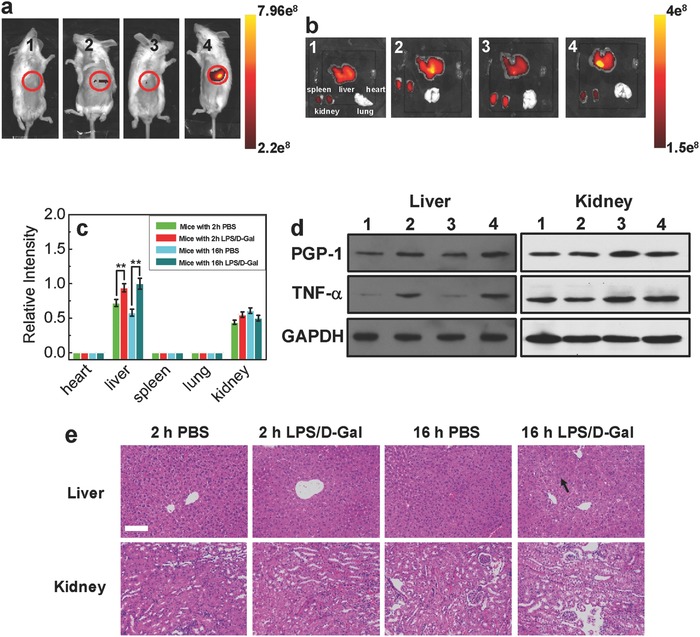
a) The fluorescence images in mice epigastrium (1 and 2: mice were first intraperitoneal injected with 100 µL of PBS and LPS/D‐Gal (40 µg kg^−1^ LPS and 400 mg kg^−1^ D‐Gal in 100 µL solution) for 2 h; 3 and 4: mice were first intraperitoneal injected with 100 µL of PBS and LPS/D‐Gal (40 µg kg^−1^ LPS and 400 mg kg^−1^ D‐Gal in 100 µL solution) for 16 h. After these, mice were intravenous injected with 100 µL of probe (50 × 10^−6^ m) in the tail vain to image). b) The fluorescence images of main organs of above mice. c) The relative fluorescent intensities of above corresponding organs (the maximum fluorescent intensity was determined to be 1, *p < 0.01, **p < 0.001, two‐sided Student's *t*‐test). d) The western blot analysis of PGP‐1 and TNF‐α in the above corresponding mice organs. e) Histopathology assessment of above mice livers and kidneys (the black arrow refers to inflammatory tissue necrosis; scale bar: 100 µm).

Finally, to further investigate the relationship between PGP‐1 and inflammation, the small RNA interference (siRNA) experiment was used to deeply explore this issue. As shown from **Figure** **4**a,b, first, it can be seen that the fluorescence of siRNA‐transfected RAW2647.4 cells is weaker than that of normal cells, which means the knock down of PGP‐1 expression in the cells, and subsequently this result is further confirmed by flow cytometry and WB analysis (see inset in Figure 4b). These all demonstrate that the probe has an outstanding selectivity to PGP‐1. More importantly, the up‐regulation of TNF‐α in siRNA‐transfected RAW264.7 cells under the stimulation LPS is feebler (Figure 4c,d). That means, inhibiting PGP‐1 with siRNA leads to the weakness of inflammatory process in RAW264.7 cells and PGP‐1 can be served as a new inflammatory cytokine.

**Figure 4:**
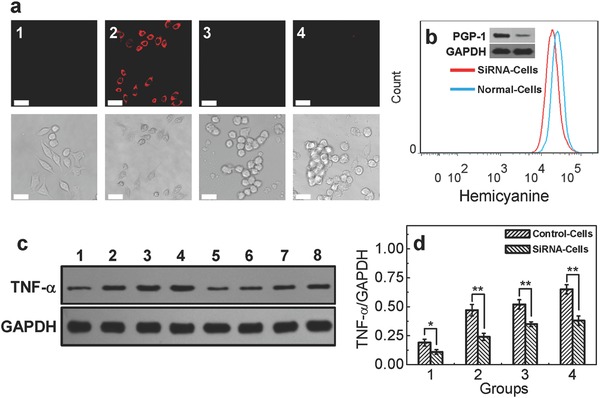
a) Fluorescence images of PGP‐1 in RAW264.7 cells. 1: normal cells only, 2: the cells were incubated with the probe (5 × 10^−6^ m) for 25 min, 3: siRNA‐transfected (100 × 10^−6^ m siRNA for 48 h) cells only, 4: the siRNA‐transfected cells were incubated with the probe (5 × 10^−6^ m) for 25 min. Scale bar: 25 µm. b) Flow cytometry results of normal and SiRNA‐transfected cells (the inset shows the WB analysis result of PGP‐1 in two kinds of cells). c) The WB analysis results of TNF‐α in RAW264.7 cells (1–4: the changes of TNF‐α in normal cells under the stimulation of LPS 0, 0.2, 0.5, and 1.0 µg mL^−1^, respectively, for 16 h; 5–8: the changes of TNF‐α in siRNA‐transfected cells under the stimulation of LPS 0, 0.2, 0.5, and 1.0 µg mL^−1^, respectively, for 16 h. d) The statistical results of above samples (**p < 0.001, as compared with the control, two‐sided Student's *t*‐test).

In conclusion, we have developed a new NIR fluorescent probe, which exhibits good selectivity and super sensitivity. Using the probe, the up‐regulation of PGP‐1 in BALB/c mice legs and livers under the stimulation of immunopotentiators is revealed for the first time, which is evidenced by WB analysis. And the occurrence of inflammatory process (including tissue necrosis) in mice is determined by up‐regulation of TNF‐α or H&E staining. Very interestingly, it is revealed that inhibiting PGP‐1 with siRNA leads to the weakness of inflammatory process in RAW264.7 cells (weak up‐regulation of TNF‐α). These important findings as well as the previous results suggest that PGP‐1 may be a new inflammatory cytokine, and provide us a new viewpoint for inflammation‐related medicinal development and disease treatment. And the relationships between some other important inflammation‐related bioactive substances or signaling molecules and PGP‐1 can be explored based on our new findings. Besides, it is believed that with our probe more in vivo biological functions of PGP‐1 can be revealed.

## Experimental Section

*Reagents*: N‐(tert‐butoxycarbonyl)‐L‐pyroglutamic acid (BOC‐L‐pyroglutamic acid) was obtained from Tokyo Chemical Industry Co., Ltd. N,N‐Diisopropylethylamine (DIPEA) and 3‐nitrophenol were purchased from Acros Organics. Stannous chloride (SnCl_2_) was obtained from J. K. Chemical Co., Ltd., Beijing, China. 2‐(7‐Aza‐1H‐benzotriazole‐1‐yl)‐1,1,3,3‐tetramethyluronium hexafluorophosphate (HATU) and trifluoroacetic acid (CF_3_COOH, HPLC grade) were purchased from Alfa Aesar Chemicals. 11‐Chloro‐1,1′‐di‐n‐propyl‐3,3,3′,3′‐tetramethyl‐10,12‐trimethyleneindatricarbocyanine iodide (IR‐780 iodide), FCA, LPS, D‐Gal, dimethyl sulfoxide (DMSO), iodoacetamide, esterase, prolidase, trypsin, leucine aminopeptidase, dipeptidyl peptidase IV, and commercial PGP‐1 probe (L‐pyroglutamic acid 7‐amido‐4‐methylcoumarin) were purchased from Sigma‐Aldrich. Fibroblast activation protein was purchased from R&D Systems Inc. PGP‐1 (molecular weight, 23 kDa) was obtained from State Key Laboratory of Antibody Medicine and Targeted Therapy, Shanghai, China. Lipidosome 3000, Roswell Park Memorial Institute‐1640 medium (RPMI‐1640), new‐born calf serum (CS), and RAW264.7 cell lines were purchased from KeyGEN BioTECH Co., Ltd., Nanjing, China. Proteins were pure as judged by Coomassie‐stained sodium dodecyl sulfate (SDS)‐polyacrylamide gel electrophoresis. Radio immunoprecipitation assay lysis buffer (CW2333) was purchased from CWbiotech Co. Ltd., Beijing, China. Agarose, poly(vinylidene fluoride) membranes, gel electrophoresis kits, WB kits, enhanced chemiluminescence kits (ECL), PGP‐1 siRNA (sequence: GGUUACAAAGGACUGGAUATTUAUCCAGUCCUUUGUAACCTT), and Braford protein assay kits were purchased from KeyGEN BioTECH Co. Ltd., Nanjing, China. Anti‐TNF‐α and Anti‐PGP‐1 antibody were purchased from Proteintech, USA. All other chemicals used were local products of analytical grade. Ultrapure water (over 18 MΩ cm) from a Milli‐Q reference system (Millipore) was used throughout. The stock solution (500 × 10^−6^ m) of probe was prepared by dissolving requisite amount of it in DMSO. Stock solutions of other substances were prepared by dissolving in PBS or water.

*Apparatus*: Fluorescence measurements were made on a Fluoromax‐4 Spectrofluorometer (France). UV–vis absorption spectra were measured in 1 × 1 cm quartz cells with a TU‐1900 spectrophotometer (China). ^1^H NMR and ^13^C NMR spectra were measured with a Bruker Avance 400 spectrometer in CD_3_OD. Electrospray ionization mass spectra (ESI‐MS) were measured with an LC‐MS 2010A (Shimadzu) instrument. High‐resolution ESI‐MS (HR‐ESI‐MS) were recorded on a TripleTof 4600 instrument (AB Sciex, USA). Cell imaging experiments were operated on a TSC SPS‐II confocal fluorescence microscope (Leica, Germany). Mice imaging experiments were operated on PerkinElmer IVIS Lumina XRMS instrument (PerkinElmer, USA). Flow cytometer results were obtained from a BD LSRFortessa flow cytometry (USA). The WB signal was detected using an ECL kit. Cytotoxicity assay was made on a microplate absorbance reader (Biorad iMARK, USA).

*Synthesis of Probe*: Hemicyanine fluorophore was obtained according to the previous method.[[qv: 5b]] BOC‐L‐Pyroglutamic acid (114.5 mg, 0.5 mmol), HATU (380 mg, 1.0 mmol), and DIPEA (1.5 mmol, 326 µL) were added to 30 mL of dichloromethane (CH_2_Cl_2_) at 0 °C. After stirring for 40 min, hemicyanine fluorophore (269 mg, 0.5 mmol) was added slowly, and the reaction mixture was further stirred at room temperature overnight. Then, the mixture was diluted with CH_2_Cl_2_, and washed three times with water (100 mL × 3). The solvent was removed by evaporation under reduced pressure, and the crude product was purified by silica gel chromatography eluted with CH_2_Cl_2_/methanol (v/v, 15/1), affording intermediate S1 as a bluish violet solid (56 mg, yield 15%). The ^1^H NMR and ^13^C NMR spectra of intermediate S1 are shown below in Figures S1 and S2 (Supporting Information), respectively. ^1^H NMR (400 MHz, CD_3_OD‐*d*_4_, δ): 8.76–8.79 (d, 1H, *J* = 12 Hz), 8.14 (s, 1H), 7.33–7.69 (m, 7H), 6.57–6.61 (d, 1H, *J* = 16 Hz), 4.36–4.39 (t, 2H), 2.42–2.80 (m, 8H), 2.08–2.11 (t, 1H), 1.93–1.99 (q, 4H), 1.83 (s, 6H), 1.45 (s, 9H), 1.07‐1.10 (t, 3H). ^13^C NMR (100 MHz, CD_3_OD, δ): 178.46, 175.37, 171.30, 160.99, 153.44, 149.47, 145.98, 142.18, 141.72, 141.54, 132.38, 128.88, 128.07, 127.34, 122.50, 118.24, 116.47, 114.43, 112.83, 105.71, 104.28, 83.31, 60.70, 50.83, 30.81, 28.77, 26.75, 23.59, 21.55, 20.98, 20.19, 10.20. HR‐ESI‐MS, calcd for C_38_H_44_N_3_O_5_ [M]^+^: *m*/*z* 622.3281; found: *m*/*z* 622.3245.

Then, the probe was prepared as follows. Trifluoroacetic acid (2.5 mL) in CH_2_Cl_2_ (2.5 mL) was added dropwise to a solution of the above intermediate S1 in 5 mL of CH_2_Cl_2_ at 0 °C, and the reaction mixture was stirred at room temperature for 3 h. The solvent was removed by evaporation under reduced pressure, and the crude product was purified by flash silica gel chromatography eluted with CH_2_Cl_2_/methanol (v/v, 10/1), affording probe as a violet solid (39 mg, yield 80%). The ^1^H NMR and ^13^C NMR spectra of intermediate S1 are shown below in Figures S3 and S4 (Supporting Information), respectively. ^1^H NMR (400 MHz, CD_3_OD‐*d*_4_, δ): 8.77–8.81 (d, 1H, *J* = 16 Hz), 8.12 (s, 1H), 7.35–7.69 (m, 7H), 6.57–6.60 (d, 1H, *J* = 12 Hz), 4.35–4.40 (m, 3H), 2.73–2.79 (d, 3H), 2.38–2.53 (m, 5H), 2.21 (s, 1H), 1.93–1.97 (t, 4H), 1.84 (s, 5H), 1.28–1.33 (d, 3H). Note that the peaks around 1.0 are grease peaks from purification.^11^ ^13^C NMR (100 MHz, CD_3_OD, δ): 180.18, 178.62, 171.90, 168.53, 160.96, 153.37, 145.81, 142.02, 141.57, 132.43, 128.83, 127.81, 127.26, 122.23, 118.03, 116.74, 114.39, 112.54, 105.80, 104.30, 61.23, 57.42, 50.65, 41.47, 31.52, 29.32, 26.75, 25.38, 24.52, 23.58, 20.99, 20.19, 10.17. HR‐ESI‐MS, calcd for C_33_H_36_N_3_O_3_ [M]^+^: *m*/*z* 522.2757; found: *m*/*z* 522.2746.

*General Procedure for PGP‐1 Detection*: Unless otherwise stated, all the fluorescence measurements were made according to the following procedure. In a test tube, 20 µL of stock solution of probe and appropriate volume of PBS were mixed, followed by addition of an appropriate volume of PGP‐1 or other substance solutions. The mixed solution was adjusted to 2 mL with PBS. After incubation at 37 °C for 25 min, the reaction solution was transferred to a quartz cell of 1 cm optical length to measure fluorescence with λ_ex/em_ = 670/700 nm (both excitation and emission slit widths were set to 2.5 nm). For absorbance measurements, the same method was used. At the same time, a blank solution without PGP‐1 was prepared and measured under the same conditions for comparison. Data are expressed as mean ± standard deviation (SD) of three separate measurements.

*Molecular Docking*: The molecular docking was carried out according to the previously describe.^12^ In short, it was carried out by SYBYL‐X 2.0. The ligand molecular was drawn using the standard parameters of SYBYL‐X, then their geometric conformations were energy minimized employing the Tripos force field for 1000 steps and Gasteiger–Huckel charges were calculated. Protein receptor was prepared using the standard way. PyMOL was used as a viewer for interaction between ligands and protein receptor.

*Cell Culture and Cytotoxicity Assay*: RAW264.7 cells were cultured in RPMI‐1640 and supplemented with 10% CS. The cells were incubated at 37 °C with 5% CO_2_. The cytotoxicity of probe was tested on cells using a standard 3‐(4,5‐dimethyl‐2‐thiazolyl)‐2,5‐diphenyl‐2‐H‐tetrazolium bromide assay (MTT assay), as described previously.^13^

*Transfection*: The transfection experiments were accomplished RAW264.7 according to the previous procedure.^14^

*Cell Imaging and Flow Cytometer Experiment*: Before cell imaging, the culture media were removed, and RAW264.7 cells (including normal and transfected) were washed using RPMI‐1640 for three times. Then, the cells were incubated with the probe (5 × 10^−6^ m) at 37 °C for 25 min in RPMI‐1640, washed with RPMI‐1640 to remove the free probe, and finally subjected to fluorescence imaging or were preserved in PBS to flow cytometer experiment.

*Mice Imaging*: All mice were purchased from the Card Vince Laboratory Animal Co. Limited (Changzhou, China) and animal experiments were approved by the animal care and use committee of the Ningbo University (Permit No. SYXK Zhe 2013‐0191). For dose experiment, female mice (BALB/c, 4 weeks) were hypodermic injected with FCA (0, 25, 50, and 100 µL, respectively) in right legs, and then fed with 7 d. For time experiment, female mice (BALB/c, 4 weeks) were hypodermic injected with FCA (100 µL) in right legs, and then fed with 0, 4, 7, and 11 d, respectively. After these, mice were then hypodermic injected with 50 µL of probe (50 × 10^−6^ m) to image. In liver inflammation experiments, female mice (BALB/c, 4 weeks) were intraperitoneal injected with PBS (100 µL) and LPS/D‐Gal (40 µg kg^−1^ LPS and 400 mg kg^−1^ D‐Gal in 100 µL solution). After 2 and 16 h, mice were intravenous injected with 100 µL probe (50 × 10^−6^ m) in the tail vain to image. Unless otherwise noted, data are expressed as mean ± standard deviation (SD) of three separate measurements.

*Histopathology Assessment of Mice Liver and Kidney*: The modeled mice were sacrificed, the livers and kidneys were preserved in 4% formalin solutions and stained with H&E for histological analysis to evaluate the inflammatory conditions.

*WB*: The cell lysates were prepared according to the previous method.[[qv: 8a]] The modeled mice were sacrificed, paws, livers, and kidneys were collected, and then the proteins were extracted. After these, the cell lysates and extracted proteins (from paws, livers, or kidneys) were placed to WB experiments and WB results were obtained according to the previous method.^4^

*Statistical Tests*: The *t* analysis was operated according to the previous method.[[qv: 8a]]

## Conflict of Interest

The authors declare no conflict of interest.

## Supporting information

Supplementary
