# Supplementary material for: A Ultrasensitive Near‐Infrared Fluorescent Probe Reveals Pyroglutamate Aminopeptidase 1 Can Be a New Inflammatory Cytokine
Source: Adv Sci (Weinh). 2018 Jan 22;5(4):1700664. doi: 10.1002/advs.201700664 (PMC5908353; doi:10.1002/advs.201700664)
Supplement: Supplementary file 1 — Supplementary [file ADVS-5-1700664-s001.pdf]

Copyright WILEY-VCH Verlag GmbH & Co. KGaA, 69469 Weinheim, Germany, 2018.

ADVANCED SCIENCE
Open Access

Supporting Information

for *Adv. Sci.,* DOI: 10.1002/advs.201700664

A Ultrasensitive Near-Infrared Fluorescent Probe Reveals Pyroglutamate Aminopeptidase 1 Can Be a New Inflammatory Cytokine

*Qiuyu Gong, Ruifen Zou, Jie Xing, Lingchao Xiang, Renshuai Zhang, and Aiguo Wu**

Supporting Information

A Ultrasensitive Near-Infrared Fluorescent Probe Reveals Pyroglutamate Aminopeptidase 1 Can be a New InflammatoryCytokine

*Qiuyu Gong[#], Ruifen Zou[#], Jie Xing, Lingchao Xiang, Renshuai Zhang and Aiguo Wu**

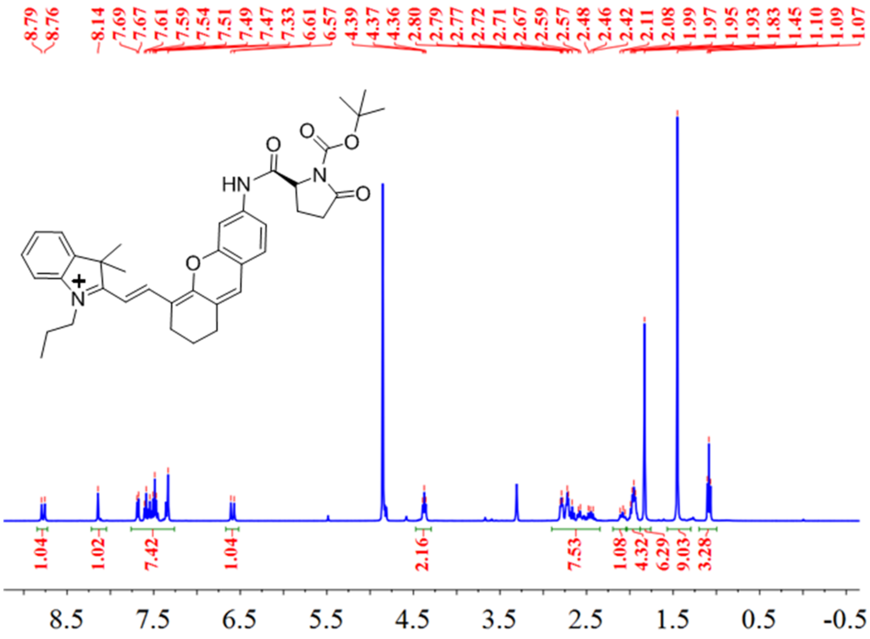

Figure S1. ^{1}H NMR spectrum of S1 (400 MHz, CD_3OD, 298 K).

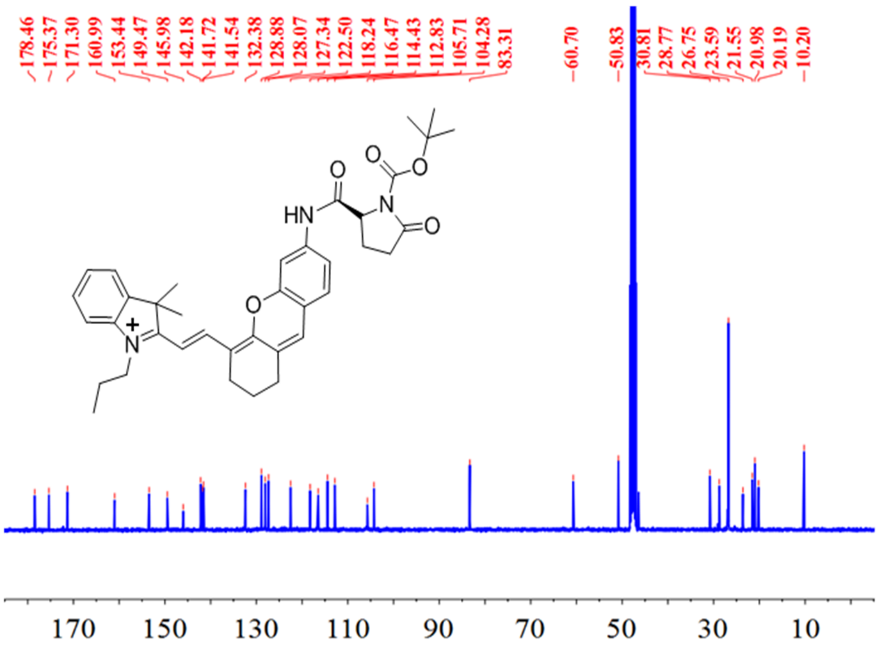

Figure S2. ^{13}C NMR spectrum of S1 (100 MHz, CD_3OD, 298 K).

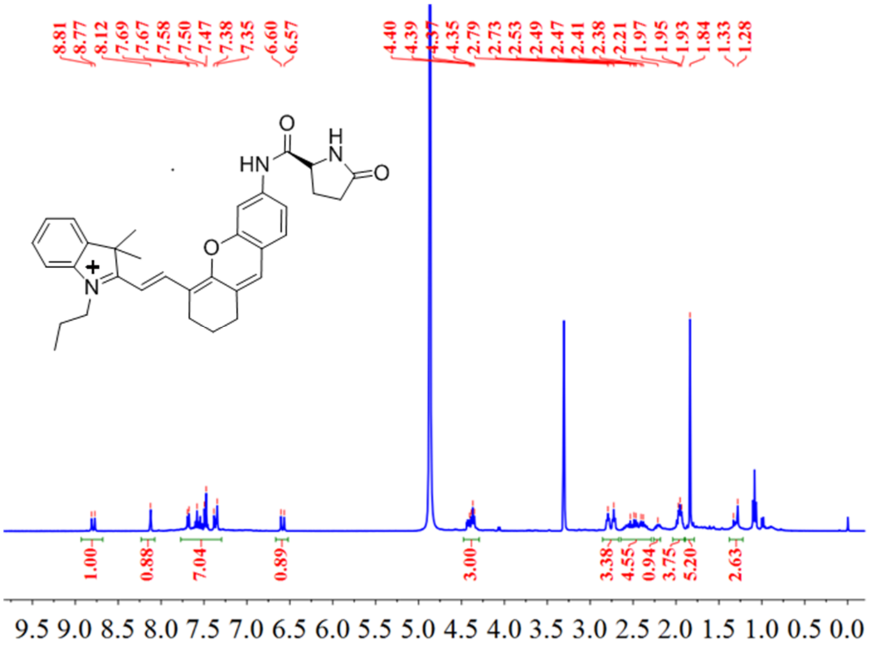

Figure S3. ^{1}H NMR spectrum of probe (400 MHz, CD_3OD, 298 K).

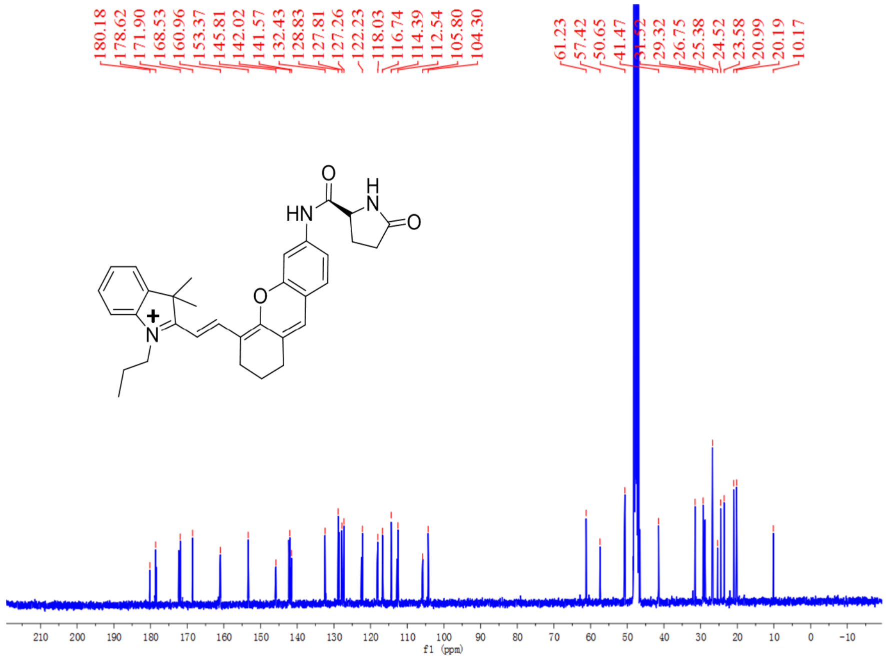

Figure S4. ^{13}C NMR spectrum of probe (100 MHz, CD_3OD, 298 K).

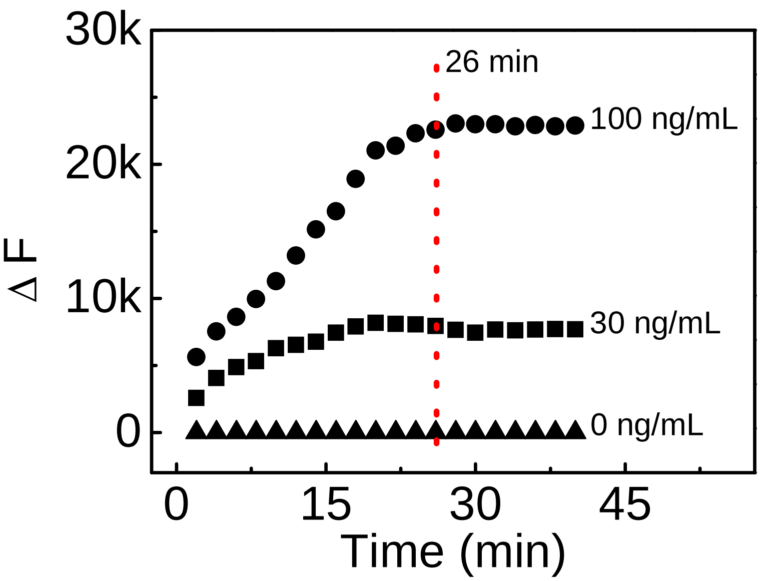

Figure S5. Effects of reaction time on the fluorescence enhancement of probe (5μM) in the presence of varied concentrations of PGP-1 (0-100 ng/mL). The reaction was performed at 37 °C in 10 mM PBS (pH 7.4). $\lambda_{ex}/_{em}$ = 670/700 nm. As

seen, a reaction time of 25 min can be chosen due to the fact that the difference of fluorescence enhancement between 25 min and 26 min is small.

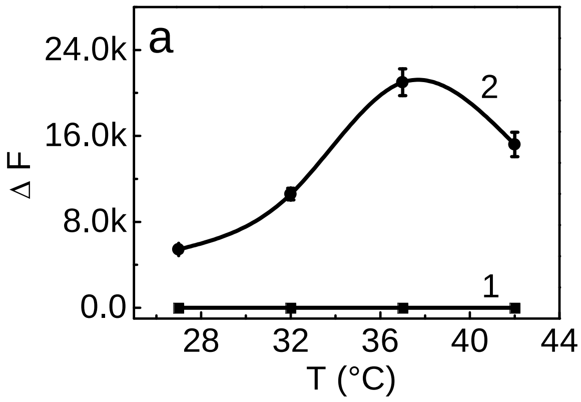

Figure S6. Effects of (a) reaction temperature and (b) pH on the fluorescence of 5 μM probe (1) without and (2) with PGP-1 (100 ng/mL). Conditions: (a) the reaction was performed in 10 mM PBS for 25 min at different pH values adjusted with dilute HCl or NaOH; (b) the reaction was performed in 10 mM PBS (pH 7.4) for 25 min at different temperatures. $\lambda_{ex}/_{em}$ = 670/700 nm. As can be seen, PGP-1 functions well under the physiological conditions (pH 7.4, 37 °C).

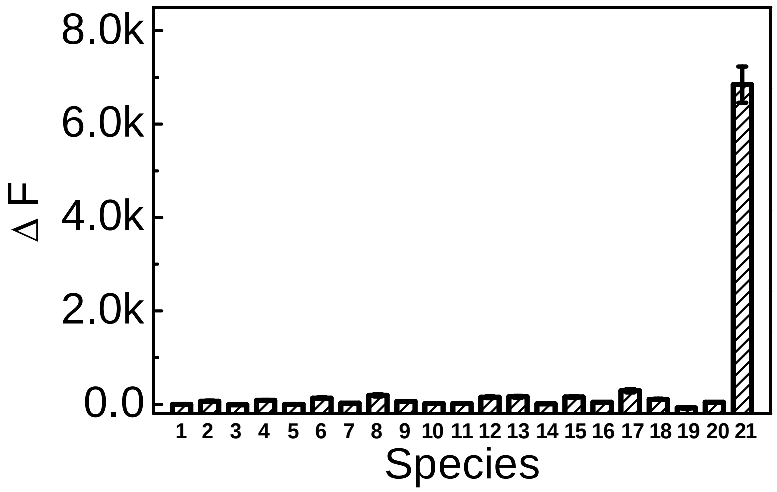

FigureS7. Fluorescence change of probe (5 μM) in the presence of various species: (1) probe only (control); (2) 150 mM KCl; (3) 2 mM $CaCl_2$; (4) 100 μM $ZnCl_2$; (5) 2 mM $MgCl_2$; (6) 100 μM $CuCl_2$; (7) 10 mM glucose; (8) 1 mM cysteine; (9) 5 mM glutathione; (10) 5 μM ClO^-; (11) 1 mM tryptophan; (12) 1 mM alanine; (13) 1 mM lysine; (14) 1 mM threonine; (15) 2 μg/mL esterase; (16) 1 μg/mL prolidase; (17) 2 μg/mL trypsin; (18) 300 ng/mL LAP; (19) 1 μg/mL FAP; (20) 300 ng/mL DPPIV; (21) 30 ng/mL PGP-1.

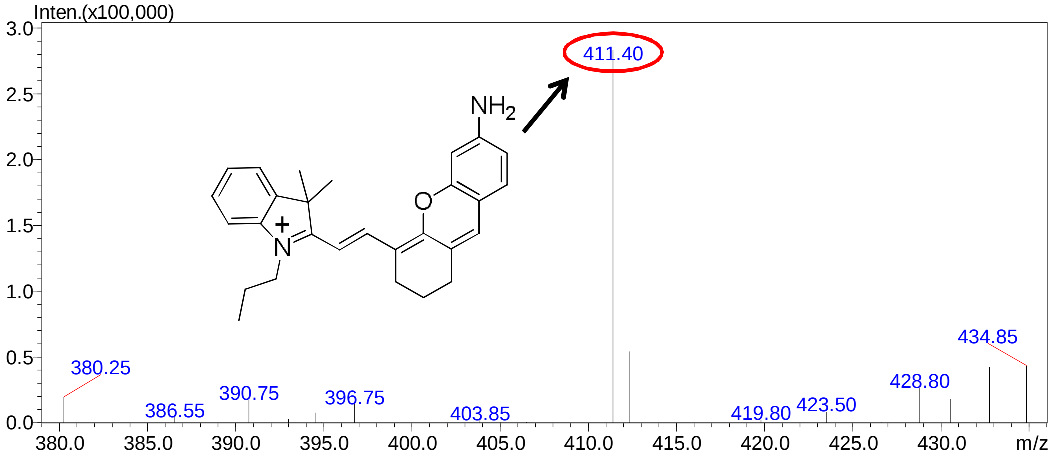

Figure S8. ESI mass spectrum of the reaction product of probe with PGP-1.

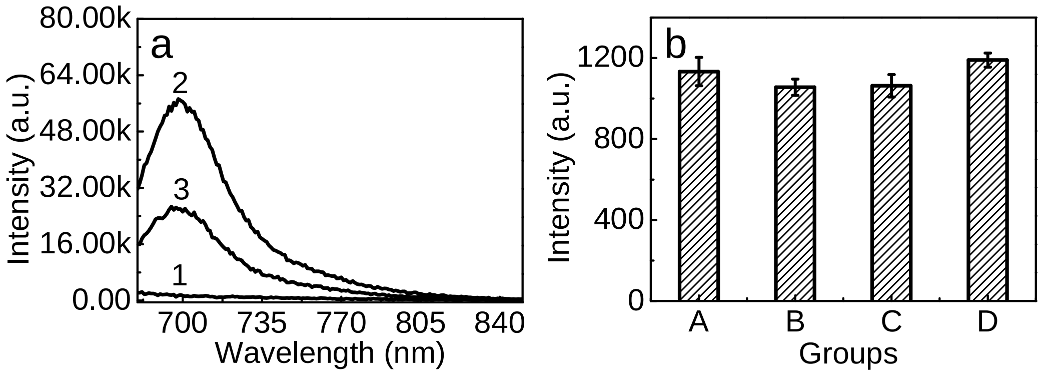

Figure S9. (a) Fluorescence emission spectra of different reaction systems. (1) probe only (5 μM); (2) probe (5 μM) + PGP-1 (300 ng/mL); (3) probe (5 μM) + PGP-1 (300 ng/mL) + iodoacetamide (100 nM). λ_{ex}= 670 nm. As is seen, addition

of the inhibitor (iodoacetamide) largely decreases the fluorescence of the reaction system via inhibiting the PGP-1 activity. (b) Effects of iodoacetamide on the fluorescent intensity of probe. A: probe only; B: A + 50 nM iodoacetamide; C: A + 100 nM iodoacetamide; D: A + 200 nM iodoacetamide.

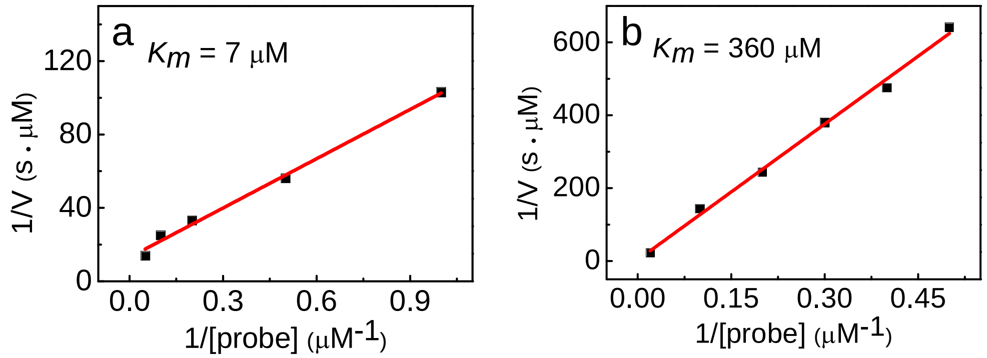

Figure S10. Lineweaver-Burk plot for the enzyme-catalyzed reaction. The Michaelis-Menten equation was described as: $V = V_{max}[\text{probe}]/(K_m+[\text{probe}])$, where V is the reaction rate, [probe] is the probe concentration, and K_m is the Michaelis constant. Conditions: (a) 100 ng/mL PGP-1, 1-20 μM probe. (b) 100 ng/mL PGP-1, 2-50 μMcommercial PGP-1 probe, pH 7.4 PBS, temperature 37 °C. $\lambda_{ex}/_{em}$ = 670/700 nm.

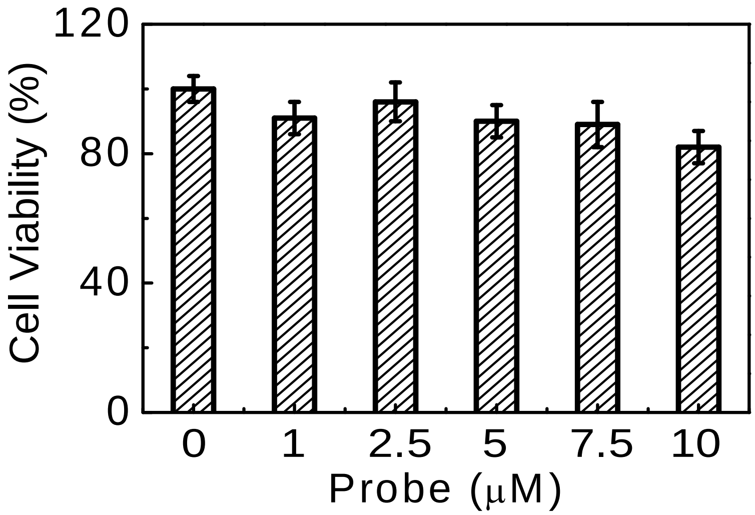

Figure S11. Effects of probe at varied concentrations on the viabilities of RAW264.7 cells.

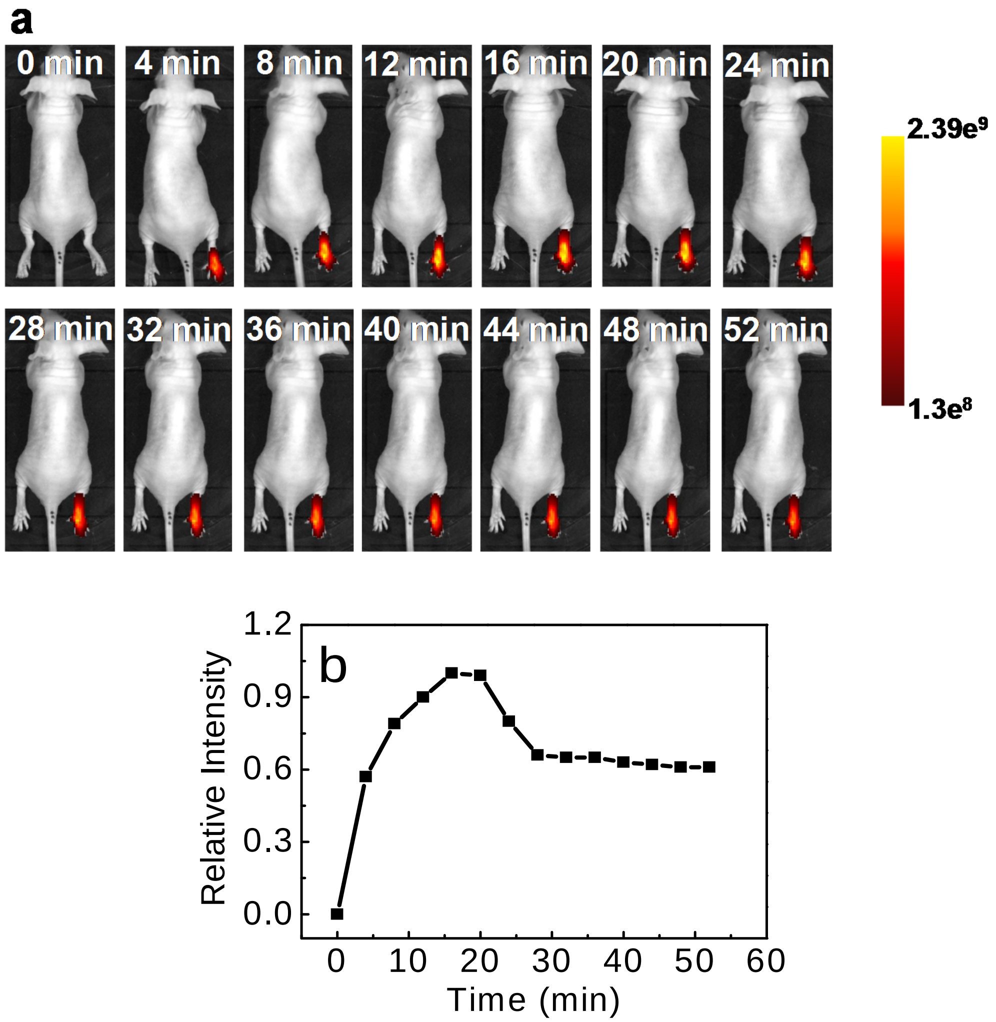

Figure S12. (a) Effects of reaction time on the fluorescence in mice legs from 0 to 52 min. Conditions: the mice were hypodermic injected with 50 µL of probe (50 µM in PBS). $\lambda_{ex}/_{em}$ = 670/710 nm. (b) Relative fluorescence intensities of above mice legs. Note that the maximum fluorescent intensity was determined to be 1.

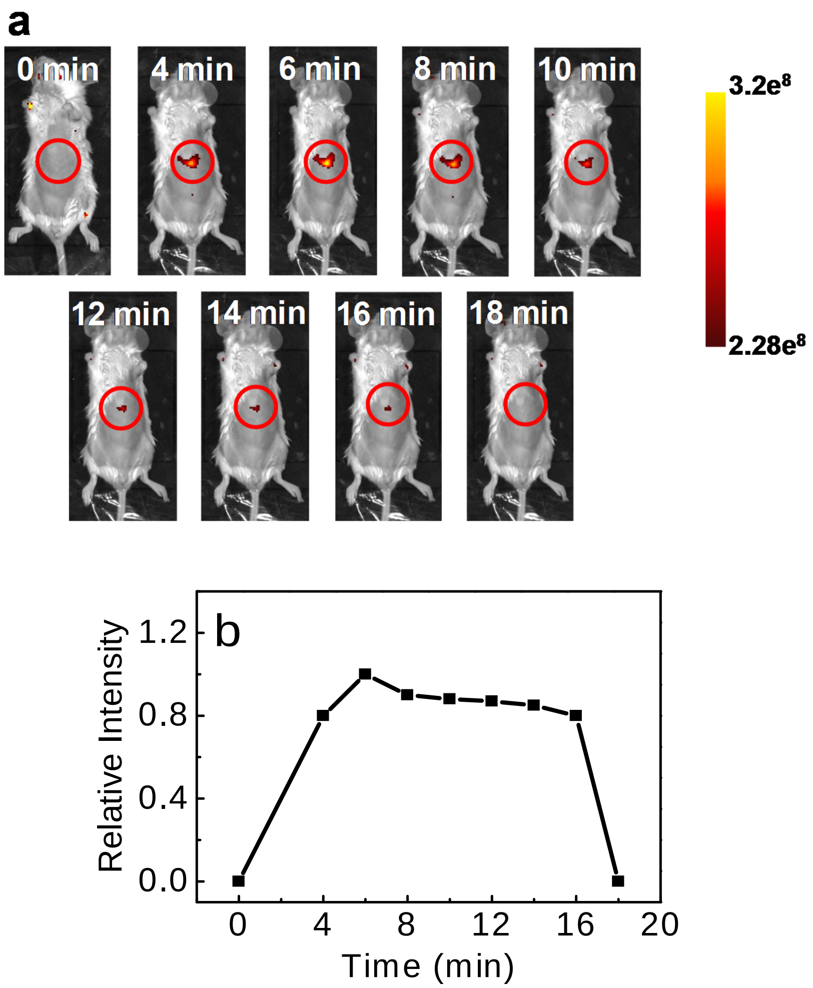

Figure S13. (a) Effects of reaction time on the fluorescence in mice epigastrium from 0 to 18 min. Conditions: the mice were first intraperitoneal injected with LPS/D-Gal (40 μg/kg LPS and 400 mg/kg D-Gal) and then feeded with 16hours. After these, mice were tail vein injected with 100 μL of probe (50 μM in PBS). $\lambda_{ex}/_{em}$ = 670/710 nm. (b) Relative fluorescence intensities of above mice epigastrium. Note that the maximum fluorescent intensity was determined to be 1.
